# The application of systems thinking in health: why use systems thinking?

**DOI:** 10.1186/1478-4505-12-51

**Published:** 2014-08-26

**Authors:** David H Peters

**Affiliations:** Department of International Health, Johns Hopkins University Bloomberg School of Public Health, Room E8527, 615 N Wolfe St, Baltimore, MD 21205 USA

**Keywords:** Complex adaptive systems, Complexity, Methods, Systems thinking, Theory, Tools

## Abstract

This paper explores the question of what systems thinking adds to the field of global health. Observing that elements of systems thinking are already common in public health research, the article discusses which of the large body of theories, methods, and tools associated with systems thinking are more useful. The paper reviews the origins of systems thinking, describing a range of the theories, methods, and tools. A common thread is the idea that the behavior of systems is governed by common principles that can be discovered and expressed. They each address problems of complexity, which is a frequent challenge in global health. The different methods and tools are suited to different types of inquiry and involve both qualitative and quantitative techniques. The paper concludes by emphasizing that explicit models used in systems thinking provide new opportunities to understand and continuously test and revise our understanding of the nature of things, including how to intervene to improve people’s health.

## Background

In the rapidly changing field of global health, it is hard to know whether the recent attention to systems thinking is just another fad, or something more durable that offers usable insights for understanding and action. Some see systems thinking as providing a powerful language to communicate and investigate complex issues, while others are confused by the sizable and amorphous body of theories, methods, and tools involved. Time will tell, of course, but in the meantime, it is helpful to consider why we would use systems thinking in a field that already draws upon a rich collection of theories, methods, and tools from the health sciences, social sciences, engineering, mathematics, and other disciplines.

## From mental models to explicit ones

At its core, systems thinking is an enterprise aimed at seeing how things are connected to each other within some notion of a whole entity. We often make connections when conducting and interpreting research, or in our professional practice when we make an intervention with an expectation of a result. Anytime we talk about how some event will turn out, whether the event is an epidemic, a war, or other social, biological, or physical process, we are invoking some mental model about how things fit together. However, rather than relying on implicit models, with hidden assumptions and no clear link to data, systems thinking deploys explicit models, with assumptions laid out that can be calibrated to data and repeated by others. The word system is derived from the Greek *sunistánai*, meaning “to cause to stand together.” If we consider that a system is a perceived whole, made up of parts that interact toward a common purpose, we recognize that the ability to perceive, and the quality of that perception, is also part of what causes a system to stand together. Systems thinking is intended to improve the quality of those perceptions of the whole, its parts, and the interactions within and between levels.

Every interpretation of a research result involves a model, whether it is a physical model used for experimentation, a statistical model used to estimate the relationships between variables, or a conceptual model about how elements are connected. A model is simply a way we compactly represent and understand an object, phenomenon, or system. As much as research involves observation and experimentation, I would argue that good research is also about building and using explicit models rather than implicit ones. The real question is not whether we should be using systems thinking, as broadly described here, but rather, which of the many theories, methods, and tools currently associated with the field of systems thinking are most useful in particular settings.

For example, where individual people interact directly with one another (e.g., transmitting disease) while moving about in an explicit space such as a city, agent-based modeling [1, 2] may be especially powerful. In modeling how different agencies within a large public health system interact, social network theory [3] could be more directly relevant.

## Origins

Systems thinking has largely developed as a field of inquiry and practice in the 20^th^ century, and has multiple origins in disciplines as varied as biology, anthropology, physics, psychology, mathematics, management, and computer science. The term is associated with a wide variety of scientists, including the biologist Ludwig von Bertalanffy who developed General System Theory; psychiatrist Ross Ashby and anthropologist Gregory Bateson who pioneered the field of cybernetics; Jay Forrester, a computer engineer who launched the field of systems dynamics; scientists at the Santa Fe Institute, such as Noble Laureates Murray Gell-Mann and Kenneth Arrow, who have helped define complex adaptive systems [4]; and a wide variety of management thinkers, including Russell Ackoff, a pioneer in operations research, and Peter Senge, who has popularized the learning organization. Much of the work in systems thinking has involved bringing together scientists from many disciplinary traditions, in many cases allowing them to transfer methods from one discipline to another (inter-disciplinarity), or to work across and between disciplinary boundaries, creating learning through a wide variety of stakeholders, including researchers and those affected by the research (trans-disciplinarity).

## Theories, methods, and tools

If there is a jungle of terminology used to describe scientific endeavor, it gets even thicker in the area of systems thinking, perhaps because of its diverse heritage. Given the varied disciplines and trans-disciplinary traditions involved, it is easy to see why people often talk about broader “approaches”, “perspectives”, or “lenses” when applying systems thinking. Systems thinking models and frameworks are sometimes grand and widely applicable, such as General System Theory, and at other times very specifically applied to particular phenomena, such as the theory on critical points in physics, which is used to explain the point at which a material behaves as neither liquid or gas (or solid). Systems thinking can involve a wide range of theories, which are rational sets of ideas or principles intended to explain something. It is based on a wide variety of scientific methods used to investigate phenomena and acquire knowledge. It uses an even larger array of instruments or tools – the hardware and software used to conduct experiments, make observations, or collect and analyze data. The use of these terms is not consistent across or within scientific fields, including systems sciences, and the continuum from tool to method to theory and framework is often blurry.

Rather than attempt to sort out semantic nuances between these terms, the utility of systems thinking can be better appreciated by a brief look at some of its more commonly used theories, methods, and tools (Table 1). The theories and methods in systems thinking are each designed to address complex problems. They are complex because they involve multiple interacting agents, the context in which they operate keeps changing, because the manner in which things change do not conform to linear or simple patterns, or because elements within the system are able to learn new things, sometimes creating new patterns as they interact over time. Many of the challenges in global health are now recognized as complex problems where simple blueprint approaches have limited success [5, 6].

**Table 1:** **Systems thinking theories, methods, and tools**

Name	Purpose and description	Key reference
**Theories**		
**Catastrophe theory**	A theory in mathematics and geometry to study how small changes in parameters of a non-linear system can lead to sudden and large changes in behavior of a system.	Poston & Stewart [7]
**Cybernetics**	Historically used as a synonym for systems theory, it is a field of study of the communication and control of regulatory feedback in both living and non-living systems (e.g., organizations, machines).	Ashby [8]
**Chaos theory**	A field of study in mathematics with applications in a wide number of disciplines to explain a dynamic system and that is highly sensitive to the initial conditions, so that small changes in initial conditions produce wildly different results. The changes occur through fixed rules about changing relationships, and without randomness.	Strogatz [9]
**General systems theory**	Less of a theory than a way of finding a general theory to explain systems in all fields of science. It was not intended to be a single theory of systems, but more of a systematic inquiry into different domains of philosophy, science, and technology.	van Bertalanffy [10]
**Learning organizations theory**	A description of organizations that facilitate learning by its members and continuously transforms itself. Systems thinking approaches are the conceptual basis for understanding the organization in its environment, and provides a basis for other key characteristics, namely a process of learning (personal mastery), the challenging and building of mental models, and the development of a shared vision and team learning.	Senge [11]
**Path dependency theories**	Occurs in economics, social sciences, and physics, and refers to the explanations for why processes can have similar starting points yet lead to different outcomes, even if they follow the same rules, and outcomes are sensitive not only to initial conditions, but also to bifurcations and choices made along the way.	Arthur [12]
**Punctuated equilibrium (in social theory)**	Theory inspired from evolutionary biology [13] to explain long periods of stasis interrupted by rapid and radical change, particularly as applied to the evolution of policy change or conflict.	Baumgartner & Jones [14]
**Methods**		
**Agent-based modeling (ABM)**	ABMs are used to create a virtual representation of a complex system, modeling individual agents who interact with each other and the environment. Although the interactions are based on simple, pre-defined rules, in a complex system these simulations allow for the identification of emergence and self-organization.	Epstein [15]
**Network Analysis (or Social Network Analysis)**	Network analysis uses graphical methods to demonstrate relations between objects. Grounded in computer science, it has applications in social, biological, and physical sciences. Social network analysis involves application of network theory to social entities (e.g., people, groups, organizations), demonstrating nodes (individual actors within a network), and ties (the type of relationships) between the actors, and uses a range of tools for displaying the networks and analyzing the nature of the relationships.	Newman [3]; Valente [16]
**Scenario planning**	This is a strategic planning method that uses a series of tools to identify and analyze possible future events and alternative possible outcomes. These can involve quantitative projections and/or qualitative judgments about alternatives. The value lies more in learning from the planning process than the actual plans or scenarios.	Schoemaker [17]
**Systems dynamics modeling**	Not a single method, but an approach that uses a set of tools to understand the behavior of complex systems over time. The methods focus on the concepts of stocks and flows and feedback loops. They are designed to solve the problem of simultaneity (mutual causation) by being able to change variables over small periods of time while allowing for feedback and various interactions and delays. The common tools include causal loop diagrams and stock and flow diagrams.	Forrester [18]
**Tools**		
**Causal loop diagrams (CLDs)**	CLDs are a system dynamics tool that produces qualitative illustrations of mental models, focused on highlighting causality and feedback loops. Feedback loops can be either reinforcing or balancing, and CLDs can help to explain the role of such loops within a given system. CLDs are often developed in a participatory approach. The drawings can be further developed by categorizing the types of variables and quantifying the relationships between variables to form a stock and flow diagram.	Williams & Hummelbrunner [19]
**Innovation (or change management) history**	Innovation or change management history aims to generate knowledge about a system by compiling a systematic history of key events, intended and unintended outcomes, and measures taken to address emergent issues. It involves in-depth interviews with as many key stakeholders as possible to build an understanding of the performance of the system from a number of different points of view.	Douthwaite & Ashby [20]
**Participatory Impact Pathways Analysis (PIPA)**	PIPA is a workshop-based approach that combines impact pathway logic models and network mapping through a process involving stakeholder engagement. PIPA workshops aim to help participants to make their assumptions and underlying mental models about how projects run explicit and to reach consensus on how to achieve impact.	Alvarez [21]
**Process mapping**	A set of tools, such as flow charts, to provide a pictorial representation of a sequence of actions and responses. Their use can be quite flexible, such as to make clear current processes, as a basis for identifying bottlenecks or inefficient steps, or to produce an ideal map of how they would like them to be.	Damelio [22]
**Stock and flow diagrams**	Stock and flow diagrams are quantitative system dynamics tools used for illustrating a system that can be used for model-based policy analysis in a simulated, dynamic environment. Stock and flow diagrams explicitly incorporate feedback to understand complex system behavior and capture non-linear dynamics.	Sterman [23]
**Systems archetypes**	Systems archetypes are a number of generic structures that describe common behaviors between the parts of a system. They provide templates to demonstrate different types of balancing and reinforcing feedback loops, which can be used by teams to come to a diagnosis about how a system is working, and particularly about how performance changes over time.	Kim [24]

Systems thinking tools have a wide variety of applications. Some tools are intended as means of facilitating groups of people to have a common understanding about an issue to prompt further inquiry and action. For example, “systems archetypes” help teams to understand generic patterns of interaction that can be applicable to their “story” [24]. Rather than use the pre-existing templates of systems archetypes, causal loop diagrams (CLD) are created without a template, and involve drawing out people’s understanding of how elements of a problem are related to each other [19]. They usually begin as qualitative descriptions outlining how one thing causes another in either a positive or negative direction. Typically, feedback loops are identified between the different elements. They can be reinforcing or positive feedback loops, where A produces more B which in turn produces more A, such as the vicious cycle of under-nutrition and infection. They can also be balancing or negative feedback loops, where a positive change in one leads to a push back in the opposite direction, such as when increasing body temperature produces sweating, which in turn cools down the body. In this supplement, a number of studies use CLDs that describe relationships between different elements of a health system to explain phenomena such as dual practice of health workers in Uganda [25], provider payment systems in Ghana [26], and childhood vaccination coverage in India [27].

The elements of a CLD might also be converted into a quantitative systems dynamics model by classifying the elements as “stocks”, “flows”, or “auxiliary” variables, and using equations to describe the relationships between individual variables in one of many available systems dynamics software environments. In this supplement, Rwashana and colleagues use systems dynamics models to examine neonatal mortality in Uganda [28], while other authors use systems dynamics models to examine the effects of policy interventions [29].

There are number of other tools that are used to map out events or how things are connected. Network mapping, social network analyses, and process mapping involve a range of tools to illustrate and analyze connections between people, organizations, or processes in both qualitative and quantitative ways. In this supplement, Malik et al. map out the network of actors involved in physician’s seeking advice in Pakistan [30]. The flow chart is one of the more common tools used to draw a process or a system. Innovation history (or change management history) is used to compile a history of key events, outcomes, issues that have cropped up along the way, and measures taken to address problems. In this supplement, Zhang et al. [31] look back over the last 35 years of the development of the medical system in rural China. Participatory Impact Pathways Analysis involves workshops and a combination of tools to clarify the logic of interventions and a mapping of the network [21]. It is intended to enhance understanding through participation with beneficiaries, implementers, and other stakeholders in a project. Several papers in this supplement use similar approaches for a variety of situations, including to build leadership capacity for health systems in South Africa [32], to develop sustainable physical rehabilitation programs in Nepal and Somaliland [33], and to build sustainable maternal and child preventive health services in Northern Bangladesh [34].

Agent based modeling takes advantage of a wide variety of theories, methods, and tools to build computer models that simulate the interaction of agents (e.g., individuals or organizations) to see how real world phenomena “grow” and affect the system as a whole. The models involve multiple individual agents that work at different scales, some decision-making rules (e.g., simple rules on how they reproduce, interact with others or pursue objectives), processes for adaptation, and a space in which the agents operate.

In global health, we are concerned with both theory and practice, and are in need of models that match the complex conditions in which we work. A common thread of all these theories, methods, and tools is the idea that the behavior of systems is governed by common principles that can be discovered and expressed. They are all helpful in trying to conceptualize the systems in place. Some are more focused on ways to change the system to produce better outcomes. In using these theories, methods, and tools, we are reminded by the statistician George EP Box that “*all models are wrong, but some are useful*” [35]. It is to these uses that we now turn.

In much of public health and medicine, we use research evidence on the efficacy of interventions to inform decisions with an expectation about their future effect. Some systems thinking methods and tools, such as scenario planning, can also be used to explicitly forecast future events. However, even then, such methods are intended to be used for identifying possible outcomes to provide insights on how to prepare for them rather than fixing on any particular outcome.

In his landmark address on “Why Model?”, which provided inspiration for this essay, Joshua Epstein identified 16 reasons other than prediction on why to model [36]. Most of these reasons are applicable to systems thinking more broadly. Many of these specific reasons relate to being able to explain how things work, and systems thinking is particularly useful to explaining how complex systems work. Many of models can be used for testing the viability of policy interventions in a safe and inexpensive way – agent based models, systems dynamics models, and scenario planning are particularly useful for these purposes. In this journal supplement, for example, Bishai et al. present a very simple systems dynamics model to illustrate the trade-offs and unintended consequences of policy choices related to allocation to preventive and curative services [29].

Systems thinking approaches can also provide guidance on where to collect more data, or to raise new questions and hypotheses. The methods and tools help us to make explicit our assumptions, identify and test hypotheses, and calibrate our models against real data. One of the frustrations of health planners and researchers has been the aspiration that interventions shown to be effective at small scale or in a research setting cannot be simply replicated at large scale or to reach populations that are most vulnerable. Systems thinking methods and tools are increasingly being used to explain epidemics and to inform programmatic expansion efforts [5, 6].

One of the more compelling reasons to use systems thinking approaches is to inspire a scientific habit of mind. Beyond the contributions of any particular theory, method, or tool, the practice of systems thinking can reinforce what Epstein calls a “militant ignorance”, or commitment to the principle that “I don’t know” as a basis for expanding scientific knowledge. Systems thinking adds to the theories methods and tools we otherwise use in global health, and provides new opportunities to understand and continuously test and revise our understanding of the nature of things, including how to intervene to improve people’s health. And for those who value thinking and doing in global health, that can only be a good thing.
